# Field-Free Superconducting Diode Effect in 45°-Twisted FeSe van der Waals Josephson Junctions

**DOI:** 10.3390/ma19050972

**Published:** 2026-03-03

**Authors:** Juyuan Wang, Wei Wei, Chuandi Pan, Hengning Wang, Chunsheng Wang, Yue Sun, Zhixiang Shi, Qun Niu, Guolin Zheng, Mingliang Tian

**Affiliations:** 1 Institutes of Physical Science and Information Technology, Anhui University, Hefei 230601, China; wangjuyuan1080@163.com; 2 Anhui Provincial Key Laboratory of Low-Energy Quantum Materials and Devices, High Magnetic Field Laboratory, Hefei Institutes of Physical Science, Chinese Academy of Sciences, Hefei 230031, China; panchuandi@mail.ustc.edu.cn (C.P.); whning@mail.ustc.edu.cn (H.W.); wangchunsheng@mail.ustc.edu.cn (C.W.); qniu@hmfl.ac.cn (Q.N.); 3 School of Physics, Southeast University, Nanjing 211189, China; 15137669720@163.com (W.W.); sunyue@seu.edu.cn (Y.S.); zxshi@seu.edu.cn (Z.S.); 4 Science Island Branch of Graduate School, University of Science and Technology of China, Hefei 230026, China; 5 School of Physics and Optoelectronics Engineering, Anhui University, Hefei 230601, China

**Keywords:** field-free superconducting diode, time-reversal symmetry breaking, iron-based superconductor, Josephson junctions

## Abstract

The iron-based superconductor FeSe has garnered considerable attention, in no small part due to its rich physics as well as the unique coexistence of superconductivity and nematicity. The recent discovery of the superconducting diode effect (SDE)—a non-reciprocal critical current with respect to the bias direction—requires simultaneous breaking of time-reversal symmetry (TRS) and inversion symmetry (IS), making it a powerful transport signature of broken symmetries in superconductors. Notably, most reported SDEs rely on the application of an external magnetic field to break TRS, which significantly limits their practical applications in integrated superconducting electronics. Here, we report a field-free SDE in 45°-twisted FeSe Josephson junctions below 3 K, evidenced directly by the even symmetric dependence of the asymmetric critical current on the magnetic field. Under temperature modulation, the SDE is progressively suppressed and ultimately exhibits a polarity reversal at 2.2 K. Our findings provide compelling transport evidence for the field-free SDE in iron-based superconductor FeSe, offering a promising platform for exploring symmetry-breaking physics and developing low-dissipation superconducting electronic devices.

## 1. Introduction

The non-reciprocal electrical transport phenomenon, characterized by asymmetric current responses under forward and reverse bias, serves as an indicator for probing symmetry breaking in condensed matter systems, thereby attracting widespread attention and intensive investigation [1,2,3,4]. The extension of this phenomenon into superconducting systems has led to the discovery of the SDE, which is typically manifested as a pronounced disparity in critical currents between opposite bias directions [5]. Recent theoretical efforts have explored the microscopic origins of SDE through non-reciprocal transport mechanisms in Josephson junctions and two-dimensional (2D) superconducting systems [6,7,8,9,10,11,12,13], while experimental observations have been realized in diverse platforms, including twisted systems [14,15,16], superconducting/magnetic heterostructures [17,18,19], thin films [20,21,22], Josephson junction devices [23,24,25,26,27], and chiral superconductors [28,29]. Notably, most reported SDEs rely on the application of external magnetic fields to achieve the necessary breaking of time-reversal symmetry, TRS, a critical constraint that significantly limits their practical applicability in integrated superconducting electronics.

Among iron-based superconductors, FeSe stands out due to its minimal crystal structure and unique coexistence of nematicity and superconducting, making it an ideal platform for studying the interplay between nematicity and superconductivity [30]. FeSe is conventionally considered to preserve both spatial-inversion and TRS symmetries [30]. However, both recent experiments such as angle-resolved photoemission spectroscopy (ARPES) [31] and scanning tunneling microscopy (STM) [32] as well as theoretical study [33] suggest the existence of hidden electronic states with TRS-breaking. Nevertheless, the corresponding transport evidence for the TRS-breaking in FeSe is still lacking.

In this work, we report the observation of a field-free superconducting diode effect in 45°-twisted FeSe van der Waals Josephson junctions. We observe a pronounced non-reciprocal current–voltage characteristic (I−V curve) in the absence of external magnetic fields. The asymmetric critical current $∆I_{c}$ exhibits an even-symmetric dependence on magnetic fields, indicating the presence of a robust field-free SDE. Temperature modulation further enables reversible switching of the diode polarity. Our results establish FeSe-based Josephson junctions as a promising platform for realizing field-free superconducting diodes.

## 2. Methods

**Figure 1:**
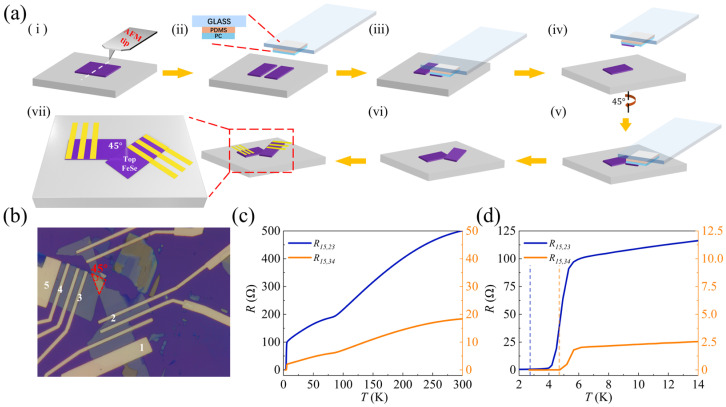
Fabrication and characterization of 45°-twisted FeSe Josephson junctions. (**a**) Schematic of the FeSe Josephson junction fabrication process. (**b**) Optical image of a typical 45°-twisted FeSe Josephson junction. (**c**) R−T curves of the bottom FeSe nanosheet and the FeSe Josephson junction, respectively. (**d**) Zoomed-in area of the R−T curves at low temperature region in (**c**). (The yellow line represents the R−T curve of the bottom FeSe nanosheet, while the blue line corresponds to that of the FeSe junctions).

The FeSe Josephson junction is fabricated via a top-down stacking approach [34], which prevents the interface from contacting any organic contaminants, thus preserving interfacial cleanliness, as illustrated in Figure 1a. FeSe was first exfoliated from the bulk crystal onto silicon substrates using scotch tapes. Nanosheets with a thickness ranging from 30 to 40 nm were selected, followed by patterning of FeSe nanosheets via the atomic force microscope (AFM) tip. Subsequently, two nanosheets were stacked away from the cut region using polydimethylsiloxane (PDMS) and polycarbonate (PC) films through a standard dry transfer technique. Finally, electron beam lithography (EBL) was carried out, and Au/Pt electrodes were deposited by sputtering. All transfer and stacking processes were performed in a glovebox with water and oxygen levels maintained below 1 ppm, utilizing a custom-built transfer stage.

During the experiments, the temperature fluctuation range of the cryostat was maintained at ±0.02 K. All low-temperature transport measurements were performed in a Quantum Design Physical Property Measurement System (PPMS) Dynacool. A focused ion beam (FIB) system with a Ga^+^ source was employed to pattern and etch the sample surface. After FIB patterning and etching, the stacked areas of devices 1, 2, and 3 were approximately 12.49 μm^2^, 37.73 μm^2^, and 42.23 μm^2^, respectively. In our experiments, a current increment of 2 μA was employed to conduct the I−V measurement.

## 3. Results and Discussion

Figure 1b illustrates the optical image of the device 1, current is applied between electrodes 1 and 5 of the device, and the voltage across electrodes 2 and 3 is recorded to obtain the resistance of the FeSe junction, denoted as $R_{15,23}$. Likewise, the voltage between electrodes 3 and 4 is recorded to obtain the resistance of the bottom FeSe nanosheet, denoted as $R_{15,34}$.

Temperature-dependent resistance curves (R−T curves) of the FeSe junction (blue, $R_{15,23}$) and the bottom FeSe nanosheet (yellow, $R_{15,34}$) are shown in Figure 1c,d, which exhibit metallic behaviors. At low-temperature regions, the superconducting transition temperature $T_{C}$, defined as the temperature at which the resistance drops to zero (as indicated by the dashed lines in Figure 1d), is 2.7 K for FeSe Josephson junction, which is much smaller than that of FeSe nanosheet ($T_{C}=4.7\;K$), as can be seen in Figure 1d. The observation of a superconducting current traversing the junction provides direct evidence for the Josephson effect in FeSe-based junctions and confirms the high quality of our devices.

**Figure 2:**
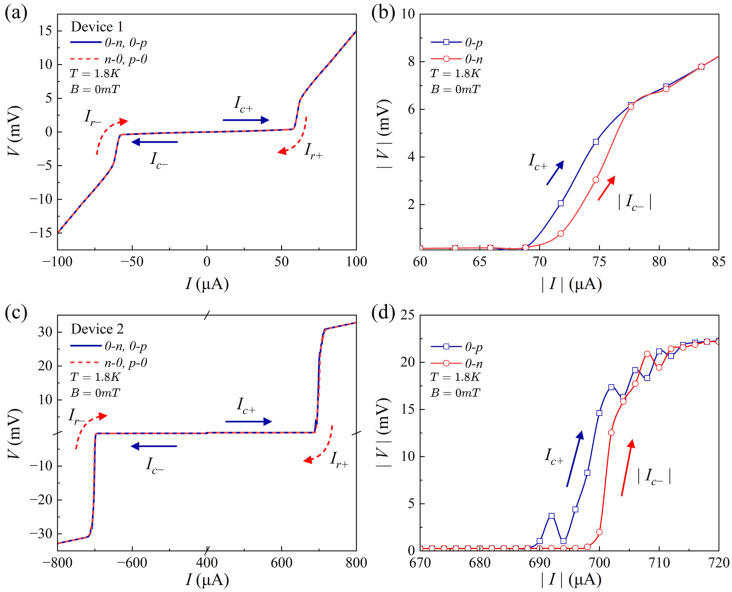
Superconducting diode effect in twisted FeSe Josephson junctions. (**a**,**c**) The voltage–current curves of device 1 (**a**) and device 2 (**c**) at 1.8 K and zero magnetic field. The blue solid line represents the critical current $I_{c\pm }$ in the forward (0−p) and reverse (0−n) sweeps, respectively. The red dashed line indicates the retrapping current $I_{r\pm }$ during forward (p−0) and reverse (n−0) sweeps, respectively. (**b**,**d**) The absolute values of I−V curves for devices 1 and 2 at positive and negative bias.

To further confirm the Josephson effect in the junctions, we measured the critical current $I_{c}$ of Josephson junctions in device 1 and device 2. We first swept the current forward from zero to positive (0−p) to obtain $I_{c+}$, and then from positive back to zero (p−0) to obtain $I_{r+}$. Similarly, in the reverse sweep, the current was swept backward from zero to negative (0−n) to obtain $I_{c-}$, and subsequently from the negative back to zero (n−0) to obtain $I_{r-}$. The $I_{c+}$ were determined to be 450 μA and 1010 μA in device 1 and 2, respectively (see Supplementary Material Figure S1a,c). To reduce the $I_{c}$ of the devices, we used focused ion beam (FIB) to etch the samples and reduce the junction areas. In addition, only the predefined sacrificial regions were exposed to ion beam irradiation, ensuring maximal protection of the devices. The I−V characteristics of the FIB-etched devices 1 and 2 are presented in Figure 2a–d.

As shown in Figure 2a,c, the values of $I_{c+}$ for device 1 and device 2 have significantly decreased to 70 μA and 690 μA, respectively. Analyzing the I−V characteristics of devices 1 and 2 reveals that $I_{r}$ and $I_{c}$ are nearly identical, indicating that the FeSe junctions exhibit characteristics of overdamped Josephson junctions. We plot the I−V curve on an absolute scale in Figure 2b,d to clearly show the asymmetric critical current ${∆I}_{c}\;$($∆I_{c}=\left|I_{c-}\right|-\left|I_{c+}\right|$), which reveals a non-reciprocal transport behavior. To quantify the SDE strength, we define the rectification coefficient $\eta =\left|\frac{I_{c+}+I_{c-}}{I_{c+}-I_{c-}}\right|$. For device 1, the measured ${∆I}_{c}$ is 2 μA, from which the η is calculated to be 1.4%, significantly higher than 0.2% obtained from the unetched FeSe junction (see Supplementary Material Figure S1b). A similar analysis for Device 2 yields a ${∆I}_{c}$ value of 8 μA and a rectification coefficient η of 0.6%. The non-reciprocal transport behaviors observed in both device 1 and device 2 reveal the existence of the SDE in the FeSe junctions. Recently, field-free SDE was reported in thermal gradient-induced FeSe nanoflakes with TRS-breaking in non-equilibrium [35]. In our experiment, however, the decrease in $I_{c}$ gives rise to the increase in η in FIB-etched samples which effectively excludes the thermal effects as the origin of the SDE (see Supplementary Material Figure S1b). Note that the I − V curve in the superconducting state for Device 1 exhibits a finite slope both before and after FIB cutting, which could be attributed to the effect of vortex dynamics. To further validate that the observed non-reciprocal behavior originates from the junction architecture rather than intrinsic properties of the material, we performed control experiments on individual FeSe nanosheets. As can be seen in Supplementary Material Figure S1d, no hysteretic I−V behavior was detected in single FeSe nanoflake, confirming that the hysteresis and associated diode effect are not inherent in FeSe nanoflakes. The emergence of non-reciprocal transport behaviors highlights the crucial role of IS-breaking in unlocking new quantum functionalities.

**Figure 3:**
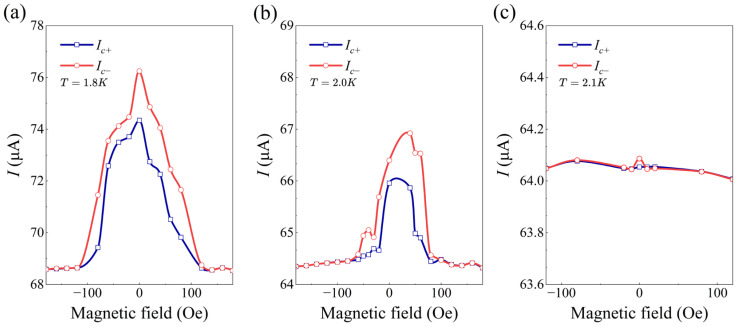
The out-of-plane magnetic field dependence of $I_{c\pm }$ in device 1 at different temperatures at 1.8=K (**a**), 2.0=K (**b**), 2.1=K (**c**). SDE exhibits a symmetric dependence on out-of-plane magnetic fields, which nearly vanishes at 2.1=K.

For a deep insight of the observed SDE, we systematically investigated the variation in the ${∆I}_{c}$ under out-of-plane magnetic fields. As shown in Figure 3a, the SDE is completely suppressed at approximately 120 Oe at T=1.8 K, indicating that the diode effect is highly sensitive to the out-of-plane magnetic field. Importantly, $I_{c}$ exhibits a clear even symmetry with respect to the magnetic field, indicating the existence of a field-free SDE with spontaneous TRS-breaking in 45°-twisted FeSe Josephson junctions. We analyze the microscopic origin of this field-free SDE and rule out vortex dynamics as the dominant mechanism, since vortex-related effects typically lead to a magnetic-field-tunable reversal of the diode polarity [15]. In contrast, the observed field-free SDE is most likely caused by spontaneous TRS-breaking intrinsic to the FeSe Josephson junctions. Figure 3b,c further demonstrate the magnetic field dependence of ${∆I}_{c}$ under different temperatures. As can be seen, the symmetry of ${∆I}_{c}$ was unchanged at different temperatures. However, in sharp contrast to the situation at 1.8 K, the critical magnetic field at which the diode effect is suppressed progressively decreases with increasing temperatures. At T=2.1 K, ${∆I}_{c}$ becomes nearly undetectable.

**Figure 4:**
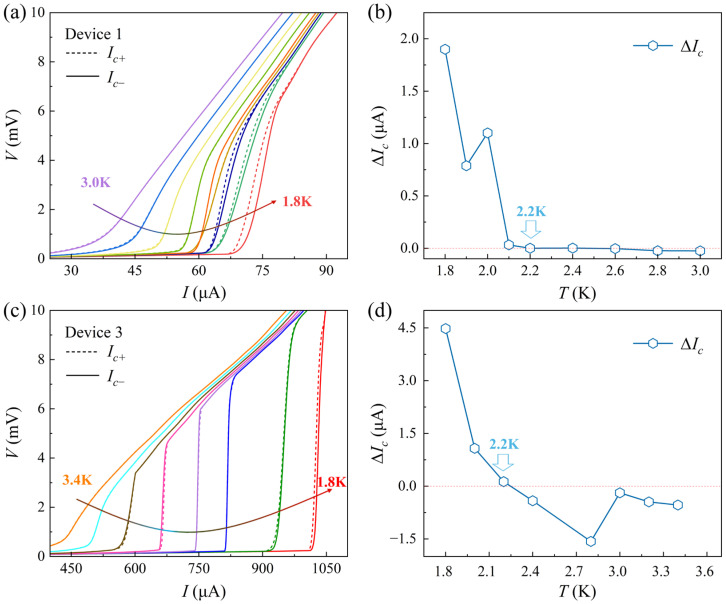
The I−V characteristics of the devices at different temperatures. (**a**,**c**) $I_{c}$ diminishes progressively with increasing temperature. (**b**,**d**) The temperature dependence of ${∆I}_{c}$ indicates the polarity reversal above 2.2=K.

We also investigated the temperature dependence of the field-free SDE, I−V characteristics of devices 1 and 3 were measured at temperatures below $T_{C}$, as shown in Figure 4a,c. As the temperature increases, $I_{c}$ decreases and ${∆I}_{c}$ is progressively suppressed. Notably, ${∆I}_{c}$ in both devices exhibit a distinct sign reversal at near 2.2 K, above which the polarity remains unchanged with further increasing temperatures. The temperature-dependent ${∆I}_{c}$ is further summarized in Figure 4b,d, which clearly illustrate sign reversals at 2.2 K, possibly due to the complex evolution of intertwined order in FeSe at low temperatures. Note that local magnetic moments of excess Fe atoms might be the underlying mechanism of TRS-breaking [36,37,38]. However, the distribution of Fe impurities varies in different nanoflakes, which cannot explain the sign reversal of SDE near 2.2 K. The observation of the field-free SDE in 45°-twisted FeSe Josephson junctions provides strong transport evidence for spontaneous TRS-breaking in iron-based superconductor FeSe.

## 4. Conclusions

In summary, the field-free SDE with an even-symmetry dependence of ${∆I}_{c}$ on the magnetic field has been observed in 45°-twisted FeSe Josephson junctions at low temperatures below 3 K. The diode polarity can be further switched by elevating the temperatures up to above 2.2 K. Our results demonstrate stable and controllable superconducting diode behavior in FeSe Josephson junctions without external magnetic fields, offering a promising platform for the design and development of symmetry-broken superconducting electronic devices.

## Data Availability

The original contributions presented in this study are included in the article/Supplementary Materials. Further inquiries can be directed to the corresponding authors.

## Supplementary Materials

The following supporting information can be downloaded at: https://www.mdpi.com/article/10.3390/ma19050972/s1, Figure S1. (a,c) The absolute values of I−V curves for unetched FeSe junctions at positive and negative bias. (b) Temperature dependence of rectification factor η in Device 1 before (blue) and after (orange) FIB etching. (d) The I−V curve of the bottom FeSe nanosheet in Device 1.
