# Supplementary material for: Field-Free Superconducting Diode Effect in 45°-Twisted FeSe van der Waals Josephson Junctions
Source: Materials (Basel). 2026 Mar 3;19(5):972. doi: 10.3390/ma19050972 (PMC12985507; doi:10.3390/ma19050972)
Supplement: Supplementary file 1 [file materials-19-00972-s001.zip › materials-4155421-supplementary.pdf]

materials

MDPI

Supplementary material

Juyuan Wang [1,2], Wei Wei [3], Chuandi Pan [2,4], Hengning Wang [2,4], Chunsheng Wang [2,4], Yue Sun [3], Zhixiang Shi [3], Qun Niu [2], Guolin Zheng [2,*] and Mingliang Tian [2,5,*]

1 Institutes of Physical Science and Information Technology, Anhui University, Hefei 230601, China; wangjuyuan1080@163.com
2 Anhui Provincial Key Laboratory of Low-Energy Quantum Materials and Devices, High Magnetic Field Laboratory, Hefei Institutes of Physical Science, Chinese Academy of Sciences, Hefei 230031, China; panchuandi@mail.ustc.edu.cn (C.P.); whning@mail.ustc.edu.cn (H.W.); wangchunsheng@mail.ustc.edu.cn (C.W.); qniu@hmfl.ac.cn (Q.N.)
3 School of Physics, Southeast University, Nanjing 211189, China; 15137669720@163.com (W.W.); sunyue@seu.edu.cn (Y.S.); zxshi@seu.edu.cn (Z.S.)
4 Science Island Branch of Graduate School, University of Science and Technology of China, Hefei 230026, China
5 School of Physics and Optoelectronics Engineering, Anhui University, Hefei 230601, China
* Correspondence: glzheng@hmfl.ac.cn (G.Z.); tianml@hmfl.ac.cn (M.T.)

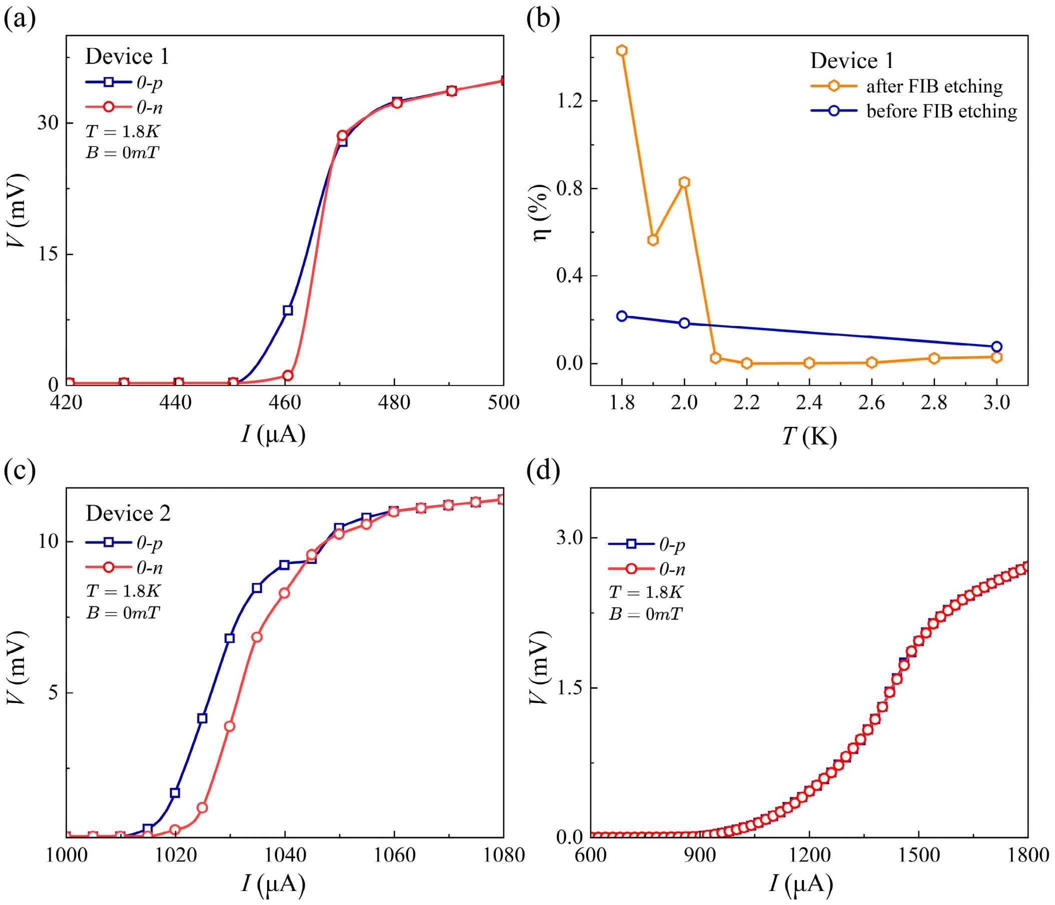

Figure S1. (a), (c) The absolute values of $I-V$ curves for unetched FeSe junctions at positive and negative bias. (b) Temperature dependence of rectification factor η in Device 1 before (blue) and after (orange) FIB etching. (d) The $I-V$ curve of the bottom FeSe nanosheet in Device 1.

Figures S1a and S1c present the $\mathrm{I}-\mathrm{V}$ characteristics of devices 1 and 2 prior to FIB processing. The measured ΔI_c values are $2\,\mu A$ and $8\,\mu A$, respectively, yielding corresponding η values of approximately 0.2% and 0.4%. Figure 1b plots the temperature dependence of the η for Device 1 before and after FIB etching. The η is significantly enhanced after FIB etching and decreases monotonically with increasing temperature. Figure 1d shows the $I-V$ characteristics of the FeSe single nanosheet obtained through forward and reverse current sweeps, I_{c+} and I_{c-} are nearly identical at $1.8\,K$ and zero magnetic field.
